# How low can you go? Antibiotic use in Swedish dogs with gastroenteritis

**DOI:** 10.3389/fvets.2024.1506106

**Published:** 2024-12-18

**Authors:** Ditte Ljungquist, Anna-Maria Andersson, Emelia Johansson, Johan Tham, Linda Toresson

**Affiliations:** ^1^Department of Translational Medicine, Lund University, Lund, Sweden; ^2^IVC Evidensia, Bristol, United Kingdom; ^3^Evidensia Sverige, Stockholm, Sweden; ^4^Faculty of Veterinary Medicine, Helsinki One Health, University of Helsinki, Helsinki, Finland

**Keywords:** antibiotic stewardship, antimicrobial resistance, canine gastroenteritis, canine diarrhea, hemorrhagic diarrhea, electronic health record, automated surveillance report, benchmarking

## Abstract

**Background:**

Canine gastroenteritis (CGE) is a common cause for seeking veterinary care in companion animal medicine and an area where antibiotics have been reported to be widely used. Therefore, creating relevant benchmarks for antibiotic use in CGE is important when implementing and analyzing antibiotic stewardship interventions. The aim of this paper was to describe the level and temporal trend of systemic antibiotic use for CGE in Sweden between 2020 and 2023.

**Materials and methods:**

This was an observational multicenter cohort study. Retrospective data from 93,641 CGE consultations was extracted from the electronic health record and analyzed. All CGE consultations were included irrespective of age, breed, severity of disease and level of care. To evaluate the data, 100 medical records of CGE consultations were also manually reviewed using a predefined study protocol.

**Results:**

The overall level of systemic antibiotic use in Swedish dogs diagnosed with gastroenteritis was 5.7% during the study period, with aminopenicillins being the most abundantly used antibiotics (60.2%). The yearly level of antibiotic use in CGE declined from 8.1% in 2020 to 3.9% in 2023, with a statistically significant annual percentage change (APC) of −21.3% (95% CI, −22.8 to −19.7). Concurrently, the annual all-cause mortality decreased for all CGE consultations. Higher levels of antibiotic use were seen in hospitalized CGE (21.7% compared to 2.1% for non-hospitalized CGE, OR 13.1, 95% CI: 12.3–14.0, *p* < 0.001) and hemorrhagic diarrhetic CGE (21.0% compared to 5.5% for non-hemorrhagic diarrhetic CGE, OR 4.6, 95% CI: 4.2–4.9, *p* < 0.001).

**Conclusion:**

This study revealed a low level and a significantly declining trend of antibiotic use in canine gastroenteritis in Sweden, implicating a high level of awareness and compliance to antibiotic guidelines among Swedish veterinarians. During the same period, the all-cause mortality rates decreased significantly for all CGE consultations, implicating that this level of antibiotic use do not compromise patient safety. Benefiting from automatic surveillance, we hereby provide important benchmarks which should encourage more prudent use of antibiotics in CGE internationally.

## Introduction

Misuse and overuse of antibiotics in human and veterinary medicine are the most considerable driving forces for rapid development of antimicrobial resistance (AMR), posing a large threat to global health (1, 2). As bacteria, including multi-resistant bacteria, are known to be shared between people and animals, a One Health approach is essential to further counteract AMR and its threat to modern medicine (3–6). In Sweden, authorities and organizations in multiple sectors have since the 1980s drawn attention to AMR and proactively worked to limit further development through education and national guidelines (7–9). The Swedish strategic program against antibiotic resistance (Strama) have reported a 43% reduction in antibiotic prescriptions in people between 1992 and 2016 (7). In the Swedish veterinary sector, antibiotic sales have significantly declined since the mid-eighties and have stabilized at a comparatively low level in recent years (10). These national long-term efforts to counteract AMR are reflected in a favorable situation compared to many other countries in terms of antibiotic resistance (11).

To enable evaluation of national and regional efforts, recognizing prescription rates and patterns is crucial. Measuring antibiotic use can be done either by manual surveys or through automatically developed systems (12–15). Automatic monitoring of antibiotic use for different diagnoses is facilitated by the usage of an implemented and integrated diagnostic coding system (16). In Sweden, assigning a diagnosis for each veterinary consultation is compulsory and a Swedish veterinary diagnostic coding system was developed in the early 1990s (16, 17). This system was during 2019–2020 replaced by a joint Swedish and Norwegian coding system called Pyramidion (17).

Gastrointestinal disorders are some of the most common causes for seeking veterinary care in companion animal medicine and an area where antibiotics are widely used (18–24). Therefore, the European Network for Optimization of Veterinary Antimicrobial Treatment (ENOVAT) has recently assessed acute canine diarrhea as an area of special interest, emphasizing the need for antibiotic stewardship interventions (25). Up-to-date international guidelines for antibiotic use, along with consensus documents on gastroenterology management and treatment, advocate non-antibiotic treatment for most acute and chronic gastrointestinal disorders (25–30). The guidelines for acute canine gastroenteritis (ACGE) and diarrhea are based on prospective randomized controlled trials from the last decade reporting non-antibiotic treatment regimens being equally good as antibiotic treatment regimens for non-septic ACGE (31–37). Despite this, studies from the same time period have reported antibiotic use to be as high as 46–71% for acute diarrhea or ACGE (20–24). Blood in stool, pyrexia, anorexia and moderate to severe disease were in the same studies associated with antibiotic treatment (20–24).

Metronidazole has been reported to be the most commonly used antibiotic for acute diarrhea or ACGE in dogs, followed by amoxicillin with clavulanic acid, penicillin and cephalosporins (21–24). Reports on antibiotic use in chronic gastroenteritis are scarce in both human and companion veterinary medicine but antibiotics reported to be most frequently used in dogs are tylosin, metronidazole and oxytetracycline (38–41).

The aim of this work was to describe the level of systemic antibiotic use and temporal trend for canine gastroenteritis in Sweden between 2020 and 2023 and thereby enable future benchmarking, i.e., using the results as an international target.

## Materials and methods

### Study design and study cohort

This was an observational multicenter cohort study including all small animal practices owned by a large cooperate group in Sweden using Provet Cloud as unified electronic health record between 1st of January 2020 and 31st of December 2023. All practices were included (first opinion practices as well as referral animal hospitals), irrespective of being part of the cooperate group during the entire or for part of the study period.

All canine consultations, assigned a diagnostic code categorized as gastroenteritis (Supplementary Data Sheet 1) during the study period, were included irrespective of age, breed, level of care and severity of disease. Both primary and follow-up visits diagnosed as gastroenteritis were included. As data was based on unique consultations, individual patients could consequently be appearing in multiple consultations during the period.

### Data extraction and variables

#### Diagnostic codes and medical data

In conjunction with each consultation, all Swedish veterinarians are obliged to register a diagnostic code in the electronic health record. In this project, diagnostic codes and relevant medical data (e.g., hospitalization or not) from each consultation during the study period were extracted and analyzed using Microsoft Power BI linked to the electronic health record during April 2024. To accommodate for the change in diagnostic system in 2019–2020, diagnostic codes from the former Swedish veterinary diagnostic coding system as well as Pyramidion were included in the data extraction.

#### Antibiotic use

All systemic antibiotic treatments (in-house use and prescriptions) were included in the data analysis. Topical antibiotic treatments were excluded. Details on antibiotic substances used were also included. As a consultation could possibly result in the use of more than one antibiotic substance, the sum of the percentages for the different antibiotics could exceed 100%.

#### Mortality rates

To evaluate the safety of plausible changes in antibiotic use, annual all-cause mortality within 14 days after the CGE consultation was analyzed. Mortality was defined as patients receiving a diagnostic code of death and/or euthanasia or alternatively being marked as deceased in the electronic health record.

### Data quality evaluation

To evaluate if the right species, diagnostic codes, antibiotic treatments, indications for treatment and antibiotic substance used were correctly picked up by the automatic surveillance report, we manually reviewed 100 medical records of CGE consultations. Using an on-line randomization tool,^1^ we randomly selected 100 CGE consultations in total (25 for each year included in the study).

### Statistical analysis

#### Diagnostic codes

**Figure 1:**
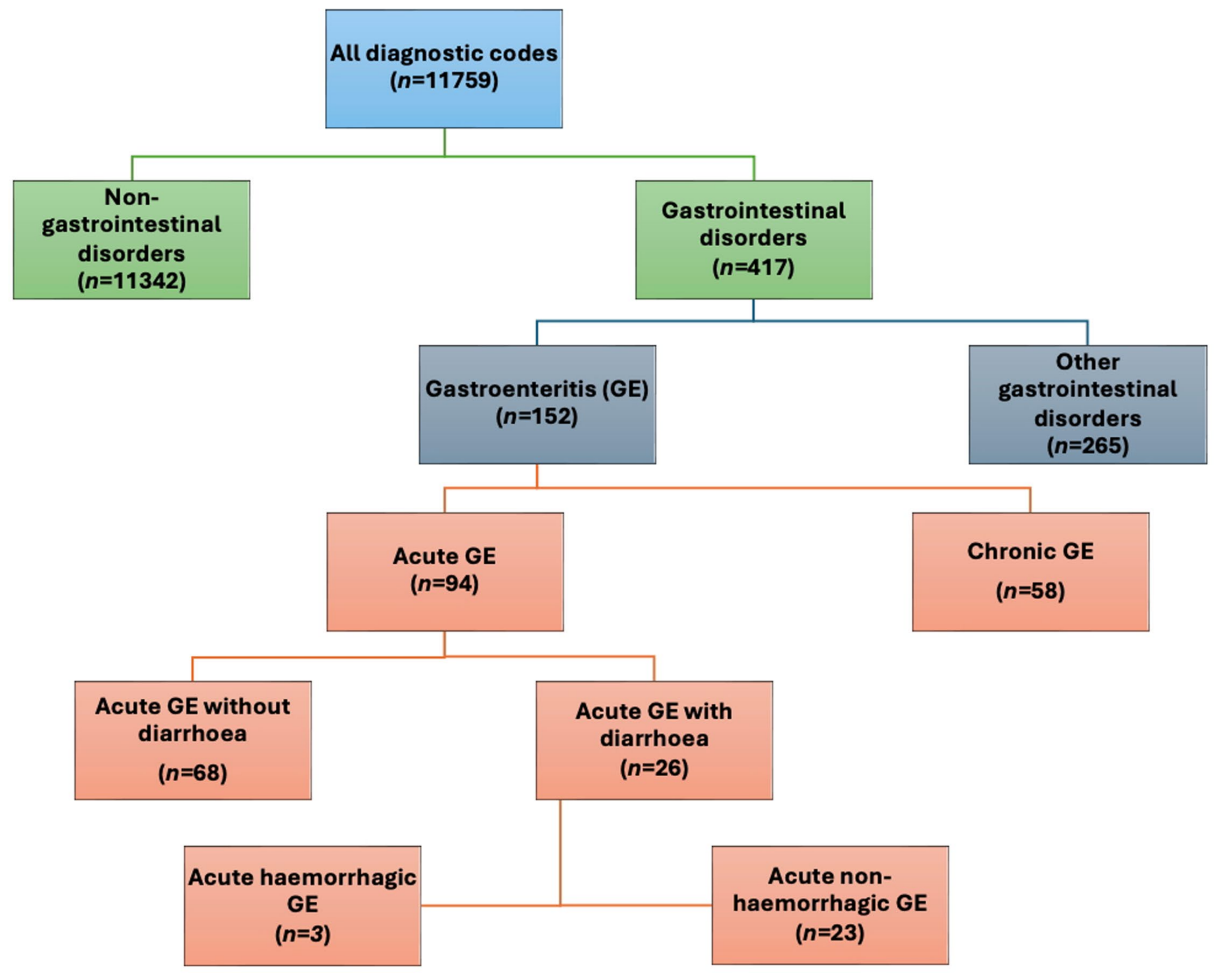
Overview of the categorisation of diagnostic codes used in this study with number of codes in each category (n=). *GE: gastroenteritis*.

To enable extraction of the study cohort from the total amount of consultations during the study period, we manually categorized all diagnostic codes used during the study period. As a first step, we separated gastroenterological disorders from non- gastroenterological disorders (Supplementary Data Sheet 1 and Figure 1). The gastroenterological disorders were then further categorized into gastroenteritis (i.e., defining the study cohort) and other gastrointestinal disorders as seen in Figure 1. Gastroenteritis codes were classified as being acute or chronic, diarrhetic or non-diarrhetic as well as hemorrhagic or non-hemorrhagic. Subcategorization of chronic CGE (CCGE) was not performed. Furthermore, the data did not allow differentiation between septic and non-septic ACGE.

Consultations classified as CGE with multiple diagnoses were only counted once. A consultation categorized as CCGE was classified as chronic regardless of other additional diagnoses.

#### Antibiotic use and mortality rates

##### Overall level of antibiotic use and per subcategory

The level of antibiotic use per consultation was summarized for CGE in general and for each sub-category of CGE separately, along with the range for different practices in the cohort (denoted as *Range_pra_*). The antibiotic use was analyzed separately for non-hospitalized and hospitalized cases for all subcategories of CGE.

Consultations resulting in in-house antibiotic treatment or prescription were counted as one antibiotic treatment, irrespective of if more than one systemic antibiotic was used or if an in-house treatment was followed by a prescription. For the different CGE subcategories, the percentages of consultations receiving the six most commonly used antibiotic groups were calculated.

Statistical significance difference in antibiotic use between the various groups was analyzed using the Chi^2^ test in the software GraphPad Prism 10.2.2 (GraphPad Software, Boston, USA). For each comparison, the odds ratio (OR) and 95% confidence interval (CI) was calculated.

##### Temporal trend

The temporal trend, expressed as annual percentage change (APC) with 95% CI, for antibiotic use and mortality rates during the study period was calculated using the Joinpoint regression program 5.0 (Joinpont trend analysis software, Calverton, USA). The predefined level of significance was *p* < 0.05. Permutation testing was used to correct for multiple comparisons.

##### Impact of practice characteristics and patient load

To evaluate the impact of practice characteristics (intensive care unit (ICU) and ward facilities) and patient load (defined as total number of GE consultations) on the antibiotic use, a fractional response regression model was fit in Stata18 (Stata Statistical Software, Texas, USA) with antibiotic use as the dependent variable. This model was chosen to accommodate for the outcome (antibiotic use for the different practices) being a fraction between zero and one. Before the regression model was fitted, possible collinearity between the different independent variables were assessed using Spearman rank correlation. Backwards elimination was used to refine the regression model based on decreased Akaikes information criterion and an exclusion of non-significant variables. The odds ratio for the dependent variables in the final model was determined.

### Ethical approval

According to Swedish regulations, no ethical approval was needed for this anonymized retrospective observational study, as the study did not interfere with routine veterinary practice (42, 43).

## Results

### Study cohort

#### Practices

In total, 65 small animal veterinary practices were included in the study cohort. Out of these, eleven were referral animal hospitals with in-patient care, and 54 were first opinion practices. Out of the referral hospitals, nine had ICU. Both first opinion practices as well as referral hospitals were represented in all major geographical regions of Sweden.

#### Consultations

During the study period, in total 1,495,284 consultations were registered for the practices included in the cohort. Out of these, 93,641 (7.4%) consultations were assigned a diagnostic code categorized as CGE. The subsequent number of CGE consultations included in the cohort were 21,799, 23,423, 23,381 and 25,038 for years 2020, 2021, 2022 and 2023, respectively.

The number of CGE consultations per practice for the entire study period ranged between 5 and 10,768 (Median: 602) depending on the size of the practice as well as the date of the onboarding to the corporate group.

### Diagnostic codes

Out of the 93,641 CGE consultations, the most frequently used CGE diagnostic codes were “Vomitus” (42%), “Diarrhea” (37.5%), “Hemorrhagic diarrhea” (7%) and “Vomitus and diarrhea” (4%). Approximately a quarter (26.9%) of the CGE consultations were assigned more than one diagnostic code classified as CGE, e.g., “Vomitus” and “Diarrhea.”

Further categorizing the CGE consultations, a majority were categorized as acute canine gastroenteritis (ACGE) (92%; *n* = 86,043; Figure 1 and Table 1). Out of the ACGE consultations, 61% (*n* = 52,424) were classified as diarrhetic ACGE (DACGE) and 39% (*n* = 33,619) as non-diarrhetic ACGE (NDACGE) with hemorrhagic DACGE cases corresponding to almost 14% (*n* = 7,190) of the DACGE cases. Hospitalization ensued for 18.4, 23.1, and 40.4% of the total CGE, DACGE and hemorrhagic DACGE consultations, respectively.

**Table 1:** Number of CGE consultations and antibiotic use for each CGE subcategory during years 2020–2023 in Sweden.

	CGE	ACGE	NDACGE	DACGE	Non-hemorrhagic DACGE	Hemorrhagic DACGE	CCGE
Number of consultations	93,641	86,043	33,619	52,424	45,234	7,190	7,598
Non-hospitalized	76,436	69,138	28,841	40,297	36,010	4,287	7,298
Hospitalized	17,205	16,905	4,778	12,127	9,224	2,903	300
Antibiotic use							
n	5,323	5,183	1,186	3,997	2,490	1,507	140
%	5.7%	6.0%	3.5%	7.6%	5.5%	21.0%	1.8%
(Range*_pra_*)	(0–13.9%)	(0–14.1%)	(0–13.5%)	(0–18.7%)	(0–13.3%)	(0–61.6%)	(0–20.0%)
Non-hospitalized							
n	1,584	1,520	405	1,115	752	363	64
%	2.1%	2.2%	1.4%	2.8%	2.1%	8.5%	0.9%
(Range*_pra_*)	(0–13.9%)	(0–13.8%)	(0–9.5%)	(0–18.4%)	(0–13.3%)	(0–61.6%)	(0–20.0%)
Hospitalized							
n	3,739	3,663	781	2,882	1738	1,144	76
%	21.7%	21.7%	16.4%	23.8%	18.8%	39.4%	25.3%
(Range*_pra_*)	(13.6–33.8%)	(0–33.6%)	(0–31.2%)	(0–38.8%)	(10.1–31.9%)	(30–64.6%)	(0–45.5%)

CGE, canine gastroenteritis; ACGE, acute canine gastroenteritis; NDACGE, Non-diarrhetic acute canine gastroenteritis; DACGE, Diarrhetic acute canine gastroenteritis; CCGE, Chronic canine gastroenteritis; Range_pra_, Practice range of antibiotic use for the included practices in the cohort.

### Data quality evaluation

According to the manual review of medical records, 97/100 (97.0%) consultations were correctly classified as CGE. Three dogs had other concurrent diseases which could possibly have been the cause of the gastrointestinal clinical signs. These dogs received a diagnostic code classified as CGE (“Vomitus” or “Diarrhea”) but also a diagnostic code relevant to the main problem (“Abdominal neoplasia,” “Adenocarcinoma” and “Pyometra”).

Among the accurately coded CGE cases, 88 out of 97 (90.7%) consultations were appropriately further subcategorized based on the clinical history. The most common misclassifications were dogs with a clinical history of hemorrhagic diarrhea (*n* = 5), receiving a diagnostic code classified as non-hemorrhagic DACGE. The second most common misclassification were dogs with a clinical history of suspected chronicity (*n* = 4), assigned a diagnostic code classified as ACGE, e.g., “diarrhea” or “vomitus.”

All treatments classifications (receiving antibiotics or not and drug of choice) for the evaluated CGE consultations were correctly coded according to the manual review.

### Antibiotic use

#### Overall level of antibiotic use in CGE consultations

For consultations assigned a CGE diagnosis between 1st of January 2020 and 31st of December 2023, the overall level of antibiotic use was 5.7% (Table 1). There was a significant difference in antibiotic use for non-hospitalized vs. hospitalized CGE cases with an antibiotic use of 2.1% for non-hospitalized dogs compared to 21.7% for hospitalized dogs (OR 13.1, 95% CI: 12.3–14.0, *p* < 0.001) (Table 1).

#### Antibiotic use in acute CGE

The level of antibiotic use in consultations classified as ACGE was 6.0% for the full study period. Out of the non-hospitalized ACGE cases, 2.2% were treated with antibiotics, whereas 21.7% of the hospitalized ACGE cases received antibiotics (OR 12.3, 95% CI: 11.6–13.1, *p* < 0.001).

Further categorizing ACGE as either NDACGE or DACGE yielded a significant difference in antibiotic use of 3.5 and 7.6%, respectively (OR 2.3, 95% CI: 2.1–2.4, *p* < 0.001). For both NDACGE and DACGE, antibiotic use differed significantly dependent on hospitalization or not. For non-hospitalized vs. hospitalized NDACGE the antibiotic use was 1.4% vs. 16.4% (OR 13.7, 95% CI: 12.1–15.6, *p* < 0.001). The corresponding figures for DACGE were 2.8%, vs. 23.8% (OR 11.0, 95% CI: 10.2–11.8, *p* < 0.001).

When splitting DACGE into hemorrhagic and non-hemorrhagic, 21.0% of the hemorrhagic DACGE received antibiotics compared to 5.5% of the non-hemorrhagic DACGE (OR 4.6, 95% CI: 4.2–4.9, *p* < 0.001). Again, the antibiotic use in non-hemorrhagic DACGE consultations that did not require hospitalization (2.1%) significantly differed from the hospitalized (18.8%) (OR 10.9, 95% CI: 10.0–11.9, *p* < 0.001). Similarly, comparing non-hospitalized and hospitalized hemorrhagic DACGE, 8.5% vs. 39.4%, were treated with antibiotics, respectively (OR 7.0, 95% CI: 6.2–8.0, *p* < 0.001). Details on antibiotic use in ACGE and subcategories are shown in Table 1 and Supplementary Data Sheet 2.

#### Antibiotic use in chronic CGE

For consultations assigned a diagnostic code categorized as chronic CGE, the overall rate of antibiotic use for the scrutinized period was 1.8%. Out of the non-hospitalized CCGE, 0.9% were treated with antibiotics whereas 25.3% of the hospitalized CCGE received antibiotics (OR 38.4 95% CI: 26.6–54.8, *p* < 0.001) (Table 1 and Supplementary Data Sheet 2).

#### Temporal trend

**Figure 2:**
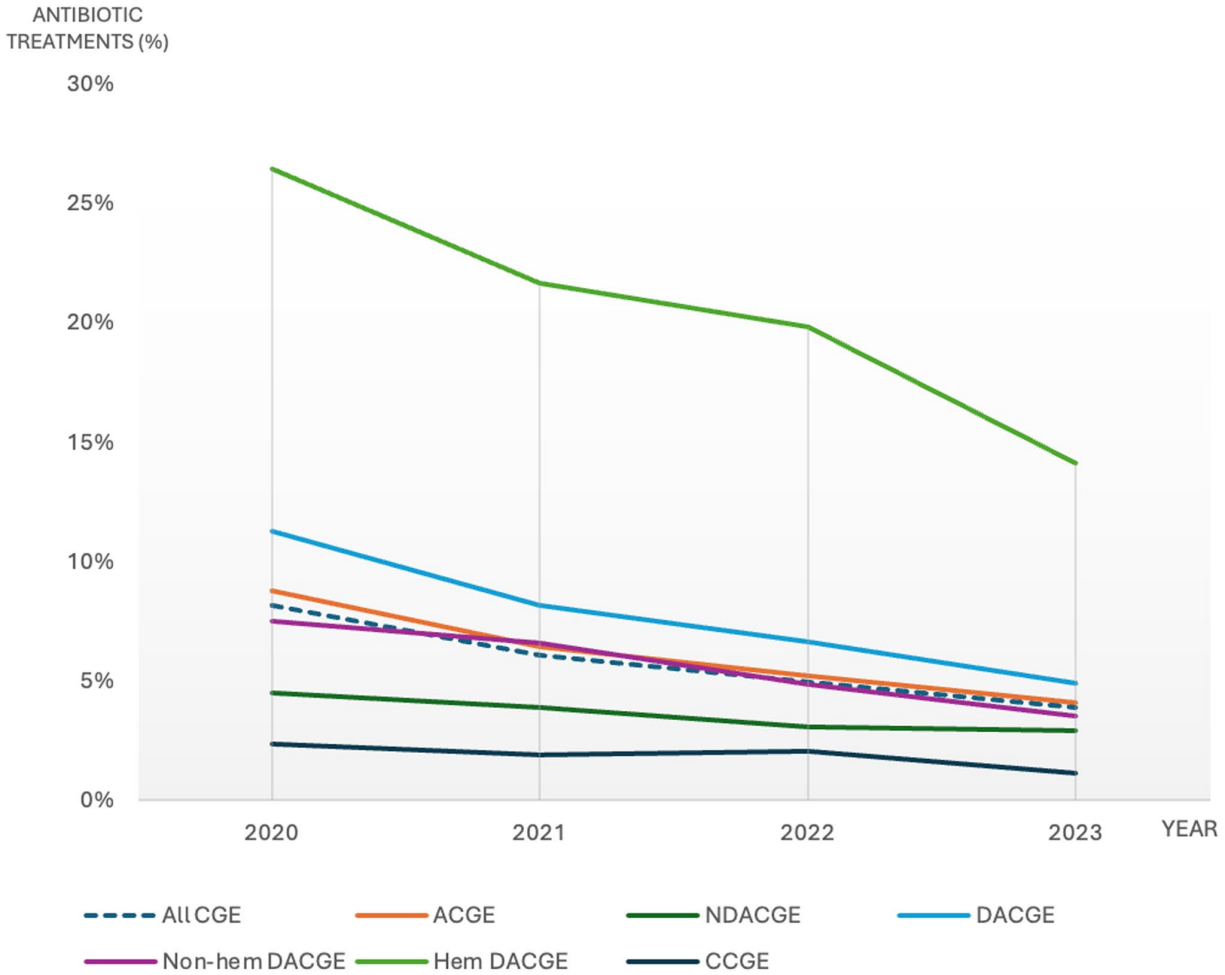
Percentage of canine gastroenteritis consultations including subcategories with antibiotic treatment during 2020-2023. *CGE: canine gastroenteritis, ACGE: acute canine gastroenteritis, NDACGE: Non-diarrhetic acute canine gastroenteritis, DACGE: Diarrhetic acute canine gastroenteritis, CCGE: Chronic canine gastroenteritis*.

There was a steadily declining trend of antibiotic use for all CGE subcategories during the study period (Figure 2 and Supplementary Data Sheet 2). The annual antibiotic use for all CGE consultations was 8.1, 6.1, 5.0 and 3.9% for years 2020, 2021, 2022 and 2023, respectively (Figure 2 and Supplementary Data Sheet 2). The Joinpoint regression model revealed a statistically significant decreasing trend in overall antibiotic use for CGE between 2020 and 2023 with an APC of −21.3% (95% CI: 19.7–22.8%).

#### Impact of practice characteristics and patient load

When evaluating the impact of practice characteristics and patient load on antibiotic use, ICU was omitted due to collinearity with in-patient care. Therefore, in the initial regression model in-patient care (yes/no) and the total number of CGE consultations in 2020–2023 were the only two independent variables included. The total number of CGE consultations was found non-significant in the initial model (*p* = 0.062) and hence not used as a predictor. Therefore, based on the final regression model, it was concluded that having in-patient care was significantly associated with an increased antibiotic use for CGE (OR 2.9, 95% CI: 1.9–4.5, *p* < 0.001). No stratified analyses were performed per CGE subcategory.

### Drug of choice

#### Antibiotics used and temporal trends in CGE

The most frequently prescribed antibiotics for CGE during the study period were aminopenicillins (without clavulanic acid) and metronidazole, followed by fluoroquinolones, potentiated sulphonamides and tetracyclines (60.2, 43.3, 3.5, 2.9% and 1.8% of the total number of CGE consultations with antibiotic treatments, respectively).

**Figure 3:**
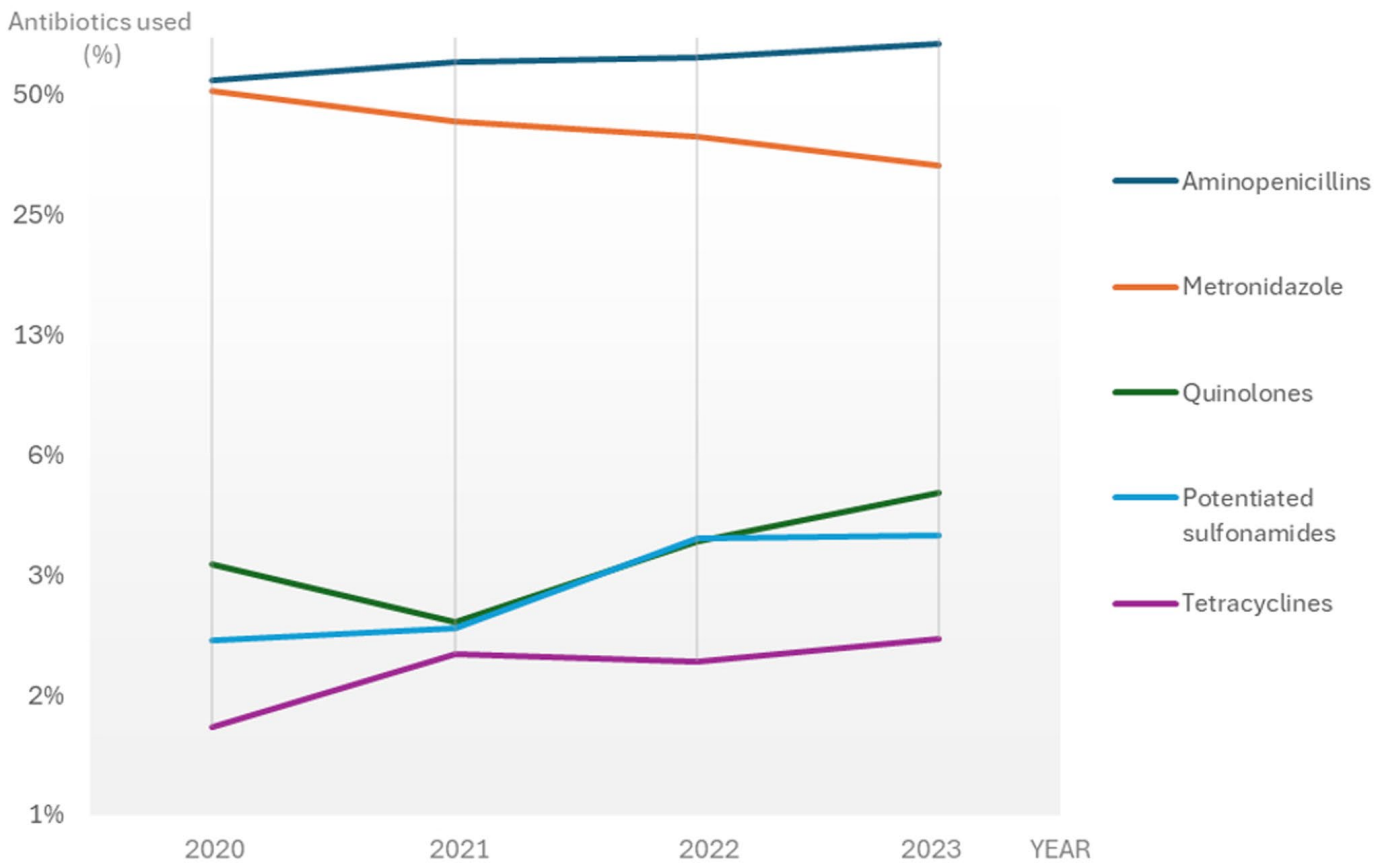
The most frequently used antibiotics for CGE consultations with antibiotic treatment in Sweden 2020-2023. A logarithmically transformed scale was used for better visualisation. *CGE: canine gastroenteritis*.

During the study period the use of aminopenicillins significantly increased from 54.6% in 2020 to 67.5% in 2023 (APC 6.9, 95% CI: 4.4–9.4%, *p* < 0.05) of the antibiotic treatments, whereas the use of metronidazole significantly declined from 51.4 to 33.3% (APC −13.0, 95% CI: −16.2% to −9.7%, *p* < 0.05) (Figure 3 and Supplementary Data Sheet 3). The use of potentiated sulphonamides and tetracyclines increased significantly with an APC of 26.3% (95% CI: 2.9–54.8%; *p* < 0.05) and 16.0% (95% CI: 0.8–33.6%, *p* < 0.05), respectively. The use of fluoroquinolones and lincosamides was not significantly altered during the study period.

#### Antibiotics used and temporal trends in CGE subcategories

**Figure 4:**
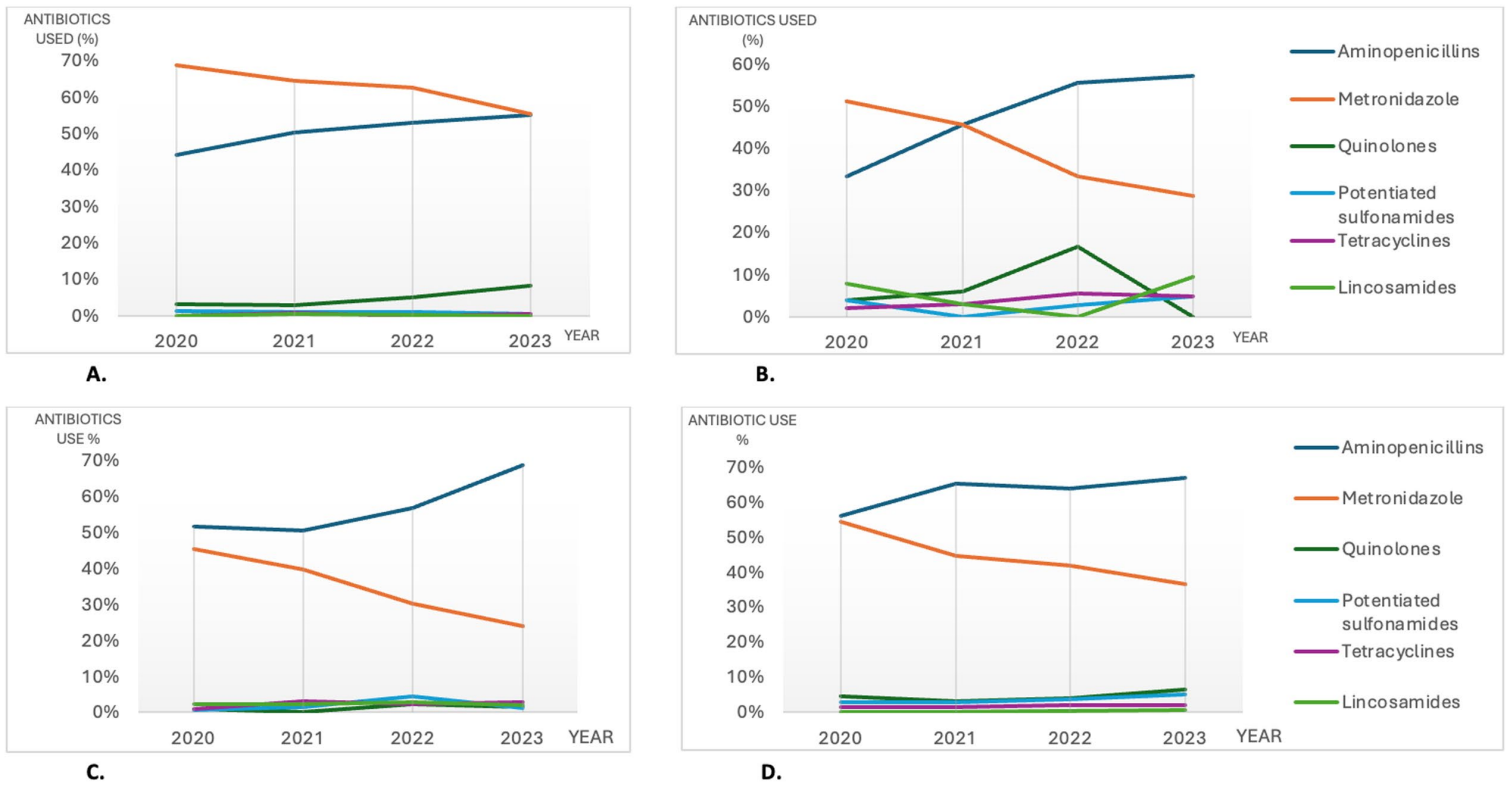
The most commonly used antibiotics among the consultations with antibiotic treatments in selected CGE subcategories (**4A**; hemorrhagic DACGE and **4B**; CCGE) and in non-hospitalized (**4C**) vs hospitalized (**4D**) CGE consultations in Sweden during 2020-2023. *CGE: canine gastroenteritis, CCGE: Chronic canine gastroenteritis, DACGE: Diarrhetic acute canine gastroenteritis, CCGE: Chronic canine gastroenteritis*.

For the entire study period, the rankings of the most commonly used antibiotics were similar for most CGE subcategories, although percentages differed somewhat between subcategories and years (details shown in Supplementary Data Sheet 3). Noteworthy, acute hemorrhagic DACGE was the only subcategory with a higher use of metronidazole (64.4%) than aminopenicillins (48.9%) of the consultations with antibiotic treatments during the study period. However, even for this subcategory there was an increase in aminopenicillin use and a decrease in metronidazole use between 2020 and 2023, resulting in comparable levels of 55.6 and 55.1%, respectively, in 2023 (Figure 4A).

In CCGE consultations with antibiotic treatments, lincosamides was the fourth most used antibiotic group (Figure 4B); a pattern that was not recognized for the other subcategories. The use of fluoroquinolones was also higher for CCGE (7.1% of the antibiotic treatments) compared to ACGE (3.4% of the antibiotic treatments) and subcategories (Figure 4B and Supplementary Data Sheet 3).

#### Antibiotics used in non-hospitalized and hospitalized CGE

When splitting CGE consultations into hospitalized and non-hospitalized, aminopenicillins and metronidazole were the most abundantly used antibiotics in both groups during 2020–2023.

Between 2020 and 2023 the use of aminopenicillins increased concurrently with a decrease of metronidazole use for both hospitalized and non-hospitalized CGE. For non-hospitalized CGE, lincosamides were the third most used antibiotics, whilst for hospitalized CGE the third most used antibiotics were fluoroquinolones. No apparent change in percentage use for these antibiotics occurred between 2020 and 2023. Details and temporal changes for the most used antibiotics are shown in Table 2 and Figures 4C,D.

**Table 2:** Antibiotics used in all, hospitalized, and non-hospitalized CGE consultations in Sweden 2020–2023.

Year		Aminopenicillins	Metronidazole	Quinolones	Potentiated sulphonamides	Tetracyclines	Lincosamides
2020	All CGE	54.6%	51.4%	3.3%	2.1%	1.3%	0.1%
	Non-hospitalized	51.7%	45.5%	1.0%	0.7%	1.0%	2.3%
	Hospitalized	56.1%	54.5%	4.5%	2.9%	1.5%	0%
2021	All CGE	60.5%	43.1%	2.4%	2.3%	2.0%	0.1%
	Non-hospitalized	50.4%	39.7%	0.1%	1.5%	3.0%	2.2%
	Hospitalized	65.3%	44.7%	3.1%	2.7%	1.3%	0.0%
2022	All CGE	62.2%	39.5%	3.8%	3.9%	1.9%	1.0%
	Non-hospitalized	56.8%	30.2%	2.2%	4.3%	2.2%	2.9%
	Hospitalized	64.0%	41.8%	3.9%	3.7%	1.8%	0.3%
2023	All CGE	67.5%	33.3%	5.1%	3.9%	2.2%	0.1%
	Non-hospitalized	68.8%	24.0%	1.6%	1.2%	2.8%	2.0%
	Hospitalized	67.1%	36.5%	6.3%	4.9%	1.9%	0.4%
2020–2023	All CGE	60.2%	43.3%	3.5%	2.9%	1.8%	0.9%
	Non-hospitalized	54.9%	38.1%	1.5%	1.6%	2.1%	2.3%
	Hospitalized	62.4%	45.5%	4.3%	3.4%	1.6%	0.3%

CGE, canine gastroenteritis.

### Mortality rates

The all-cause national mortality rate at/or within 14 days following any type of veterinary consultation within the corporate group was 1.6% for the study period. In comparison, the all-cause mortality rate for CGE consultations was 8.1% for the whole study period with a decreasing trend during the years 2020–2023 (Table 3). When scrutinizing the various subcategories, mortality rates were lowest among non-hemorrhagic DACGE consultations (6.6%) and highest among hemorrhagic DACGE consultations (8.1%) during the study period.

**Table 3:** Mortality rates at/or within 14 days following the CGE consultations (in total and per subcategory) between years 2020–2023 in Sweden (*n* = number of patients registered as deceased at/or within 14 days after a CGE consultation, *N* = total number of CGE consultations).

	All CGE	ACGE	NDACGE	DACGE	Non-hemorrhagic DACGE	Hemorrhagic DACGE	CCGE
2020							
n/N	1909/21799	1612/19635	662/7126	950/12509	733/10026	177/2483	297/2164
%	8.8%	8.2%	9.3%	7.6%	7.3%	7.1%	13.7%
2021							
n/N	1879/23423	1624/21656	762/8689	862/12967	751/11642	111/1325	255/1767
%	8.0%	7.5%	8.8%	6.6%	6.5%	8.4%	14.4%
2022							
n/N	1917/23381	1665/21599	804/8766	861/12833	737/11265	124/1568	252/1782
%	8.2%	7.7%	9.2%	6.7%	6.5%	7.9%	14.1%
2023							
n/N	1900/25038	1702/23153	808/9038	894/14115	724/12301	170/1814	198/1885
%	7.6%	7.4%	8.9%	6.3%	5.9%	9.4%	10.5%

CGE, canine gastroenteritis; ACGE, acute canine gastroenteritis; NDACGE, Non-diarrhetic acute canine gastroenteritis; DACGE, Diarrhetic acute canine gastroenteritis; CCGE, Chronic canine gastroenteritis.

The Joinpoint regression model revealed a statistically significant decrease in all-cause mortality rate for all CGE consultations with an APC of −4.1% (95% CI: −7.7% to −0.25%, *p* < 0.05). For the subcategories ACGE, DACGE, and non-hemorrhagic DACGE, there was a significant decrease in mortality rates with APCs of −2.8% (95% CI: −5.4% to −0.14%, *p* < 0.05), −5.3% (95% CI: −8.8% to −1.8%, *p* < 0.05), and − 6.2% (95% CI: −9.6% to −2.6%, *p* < 0.05), respectively. The all-cause mortality rate did not significantly alter during the study period for NDACGE, hemorrhagic DACGE and CCGE.

## Discussion

The aim of this study was to describe the levels and temporal trends of systemic antibiotic use in CGE in Sweden between 2020 and 2023. We found considerably lower levels of antibiotic use for CGE (5.7%) overall and acute diarrhetic CGE (7.6%) compared to previous international studies reporting antibiotic use of 46–71% (20–24). We also found a statistically significant annual decrease in antibiotic use for the cohort with an APC of −21.8% during the study period. Despite this, the annual mortality rates remained stable and even decreased for half of the CGE subcategories during the study period, illustrating the safety of reducing antibiotic use for CGE.

The low level of antibiotic use indicates a general high compliance to up-to-date guidelines and treatment recommendations for CGE among Swedish veterinarians. The reasons for the declining temporal trend of antibiotic use reported in this study cannot fully be explained, but may be influenced by the results from previously published prospective studies, revealing non-antibiotic treatment regimens equivalent to antibiotic treatment in non-septic CGE or diarrhea (31, 35, 36, 44).

Furthermore, there is high level of awareness regarding AMR among Swedish veterinarians, which alongside efforts of the larger corporate groups’ rigorous antibiotic stewardship programs is likely to be reflected in the results (12).

Our study encompassed mortality rates which, from an antibiotic stewardship perspective, is of great importance when using results to safely implement new strategies and guidelines. The corporate group has during the last years put an increased effort to ensure prudent use of antibiotics and is reporting a continuous decrease in antibiotic use (12). Our results from this study and other international studies from human and veterinary medicine further underline the importance and safety of implementing antibiotic stewardship programs and strategies to combat antimicrobial resistance (45, 46).

As expected, dogs that were hospitalized due to CGE received antibiotics to a higher extent than the non-hospitalized dogs in all subcategories. This is likely reflecting that severity of gastrointestinal disease influences veterinarians’ decisions to treat with antibiotics. This was also in accordance with the results from the multinomial regression model, where in-patient care was a significant predictable variable. Furthermore, the use of antibiotics was almost fourfold higher in hemorrhagic DACGE compared to non-hemorrhagic DCGE, indicating an apparent association between blood in stool and antibiotic treatment in CGE. This is in accordance with what has been concluded in other international studies and is most likely reflecting that bloody stool is considered a higher grade of severity of the disease among veterinarians in general (20, 21). However, previous prospective studies questioned the causality between blood in stool and antibiotic treatment, as there were no significant differences observed in duration of clinical signs and mortality rates for dogs treated with antibiotics or not (36, 47, 48). Furthermore, according to Swedish veterinary antibiotic guidelines, hemorrhagic CGE is not an indication for antibiotic treatment unless the dog shows signs of sepsis, has a parvoviral infection or is irresponsive to supportive care for more than 5 days (27, 28). The comparatively high level of antibiotic use in hemorrhagic DACGE might illustrate that improvements in antibiotic use for these dogs could be possible. However, since no distinction between septic and non-septic CGE or duration of clinical signs was made in this study, further evaluation of the compliance to antibiotic guidelines could not be done. Future studies on hemorrhagic DACGE are therefore warranted.

In recent years, the previously recommended antibiotic trial in dogs with chronic inflammatory enteropathies (CIEs) that do not respond to a dietary trial has been questioned and is no longer recommended by several leading gastroenterologists (29, 30). Therefore, it has been proposed that the classification of CIEs should be updated and the term antibiotic responsive enteropathy (ARE) be replaced by microbiota-related modulation-responsive enteropathy (MrMRE) (49). In this study, the level of antibiotic use for CCGE was markedly lower (1.8%) compared to all CGE consultations. We hypothesize that this reflects Swedish veterinarians’ modern approach to management of CCGE, focusing more on multiple dietary trials, microbiota-modulating treatments (prebiotics, probiotics and fecal microbiota transplantation), and immunomodulatory treatments (29, 30, 50).

In this study, aminopenicillins were the most commonly used antibiotics for all subcategories of CGE except for hemorrhagic diarrhetic DCGE, in which metronidazole was the most abundantly used antibiotic. National and international antibiotic guidelines promote primarily aminopenicillins in CGE cases otherwise fulfilling criteria for antibiotic treatment (25–28). The increasing use of aminopenicillins and declining metronidazole use in this study is probably reflecting increasing compliance to guidelines among Swedish veterinarians. However, as metronidazole is not first-hand recommendation for the few indications when antibiotic treatment is indicated, there is plausible room for improvement regarding choice of antibiotic substance. The historically high level of metronidazole use can possibly be due to the drugs proposed intestinal anti-inflammatory properties as well as its effectiveness on anaerobic bacteria (51). The role of anaerobic bacteria such as *Clostridium* spp. and other bacteria previously assessed as enteropathogenic have been questioned lately which may further have decreased the overall antibiotic use as well as the use of metronidazole (52, 53).

For the lesser used antibiotic substances, there were some notable observations. The use of potentiated aminopenicillins, mainly amoxicillin potentiated with clavulanic acid, in this cohort was trivial (data not shown). This is in contrast to previous studies from other countries and highlights the international differences in antibiotic use patterns and antibiotic stewardship efforts. Secondly, the fluoroquinolone use for CGE remained at a low level throughout the entire study period (yearly average of 3.5%). The veterinary use of fluoroquinolones is firmly regulated in the Swedish legislation and is, according to Swedish national guidelines, recommended solely for CGE cases in severe sepsis or chronic cases of granulomatous colitis (27, 54, 55). Again, as no discrepancy was made between septic and non-septic CGE consultations, the correlation between fluoroquinolone use and sepsis could not be evaluated in this study. Thirdly, there was a statistically significant increase in the use of potentiated sulphonamides. This might reflect a general increase in the use of potentiated sulphonamides for treatment of infections caused by Gram negative bacteria such as *Escherichia coli* in among Swedish veterinarians, in accordance with national and international antibiotic guidelines (25, 27).

For CCGE, the number of consultations was relatively low and hence, every prescription had the ability to impact the percentual use markedly (Supplementary Data Sheet 2 and Supplementary Data Sheet 3). The percentual use of fluoroquinolones for CCGE was fluctuating during the study period ranging from 0–16.7% and numbers were highly influenced by a few cases diagnosed with granulomatous colitis (*data not shown*). In contrast to the other CGE subcategories, the third most used antibiotic for CCGE was lincosamides. As atopic dermatitis, with consequent secondary pyoderma, and chronic enteropathies usually occur concurrently, the correlation between CCGE and a higher proportional level of lincosamide use was not surprising (56).

Our study has several strengths. It included a sizable cohort with a variability of practice size, level of care and a diverse national geographical setting. The large cohort included cases from first opinion practices as well as intensive care units and should therefore give a representative picture of the prescription behavior for CGE at all levels of care in Sweden.

There are some limitations to our study. Firstly, as this was a retrospective observational study, some limitations are inherent to the study design and all preferred variables could not be extracted, such as distinguishing septic from non-septic cases of CGE. Secondly, another source of bias when using an automatic surveillance report based on diagnostic coding, is the risk of misclassification of diagnostic codes and hence the distribution of antibiotic use. The manual evaluation of 100 medical records however, revealed a 97.0% accuracy for CGE classification and a 90.7% accuracy for the CGE subcategorization. According to the manual evaluation, the most common misclassification of the CGE subcategories was hemorrhagic DACGEs being classified as non-hemorrhagic DACGE. As hemorrhagic DACGE, according to our results, were to a much higher extent treated with antibiotics (21%) than non-hemorrhagic DACGE (5.5%), the association between blood in stool and antibiotic treatment for antibiotic use is probably slightly overestimated for hemorrhagic DACGE. Thirdly, concurrent diagnoses might also have overestimated the use of antibiotics for the CGE cohort, as the possible concurrent diseases might be the primary indication for initiating antibiotic treatment. However, as the results of this study have provided a reasonable benchmark for prudent antibiotic use in CGE, a slight overestimation of antibiotic use can be considered of less importance. Fourthly, the level of national infection prevention in Sweden is high compared to other countries (57). The high share of dogs vaccinated against canine parvovirus may influence the overall severity of disease in the cohort and hence the antibiotic use. This might influence the international generalizability of the study but also highlights the need for preventive measures to increase animal welfare internationally and reduce antibiotic use.

Identifying specific conditions that can be handled solely with non-antibiotic treatment regimens, such as specific subcategories of CGE, is one of the major cores of antibiotic stewardship (58). The declining trend of antibiotic use cannot fully be explained by this study but increasing veterinary knowledge and implementation of the corporate group’s rigorous antibiotic stewardship program are possible influencing causes.

In addition, the consistent annual decrease of antibiotic use in CGE during the study period could indicate that further declination is possible for dogs diagnosed with CGE. Our results suggests that there has been an overuse of antibiotics for CGE in Sweden, implicating an even more noteworthy overuse internationally. If the exact benchmarks concluded in this study are generalizable to other settings and populations remains to be seen. What may be concluded though is that more work must be done internationally to decrease unnecessary use of antibiotics in CGE through education and guidelines.

Future, and preferably prospective, studies would help to increase the understanding of veterinary decision making in CGE as well as finding specific areas where veterinarians deviate from the treatment recommendations. In a situation where optimized compliance to guidelines is achieved, the temporal curve would flatten and remain at a stable level until novel guidelines are presented. A follow up study for this cohort with uniform settings would be valuable to enable visualization of the future temporal trends, and hence the approximation of adherence to guidelines.

## Conclusion

This study revealed a low level and a significantly declining trend of antibiotic use in canine gastroenteritis in Sweden, implicating a high level of AMR awareness and compliance to antibiotic guidelines among Swedish veterinarians. During the same period, the all-cause mortality rates decreased significantly for all CGE consultations, implicating that this level of antibiotic use do not compromise patient safety. Benefiting from automatic surveillance, including almost a hundred thousand canine gastroenteritis consultations, we hereby provide important benchmarks which should encourage more prudent use of antibiotics in CGE internationally.

## Data Availability

The datasets presented in this article are not readily available because the raw data originates from a database subject to legal protections for intellectual property and subject to GDPR regulations. Requests to access the datasets should be directed to ditte.ljungquist@med.lu.se.

## Footnotes

^1^ random.org

## Supplementary material

SUPPLEMENTARY DATA SHEET 1 Categorization of all gastrointestinal diagnostic codes used in Pyramidion and the former Swedish veterinary diagnostic coding system.

SUPPLEMENTARY DATA SHEET 2 Number of CGE consultations (including subcategories) and level of antibiotic use per year 2020–2023.

SUPPLEMENTARY DATA SHEET 3 Antibiotic substances used in CGE consultations (including subcategories) year 2020–2023.
