# Supplementary material for: How low can you go? Antibiotic use in Swedish dogs with gastroenteritis
Source: Front Vet Sci. 2024 Dec 18;11:1506106. doi: 10.3389/fvets.2024.1506106 (PMC11688813; doi:10.3389/fvets.2024.1506106)
Supplement: SUPPLEMENTARY DATA SHEET 1 — Categorization of all gastrointestinal diagnostic codes used in Pyramidion and the former Swedish veterinary diagnostic coding system. [file Data_Sheet_1.PDF]

Code in Pyramidion or former Diagnostic coding system	Text code (Swedish) in Pyramidion or former Diagnostic coding system	Gastro-enteritis (yes=1)	Acute gastro-enteritis (yes=1)	Diarrhetic gastro-enteritis (yes=1)	Hemorrhagic (yes=1)
DB	Magsäck, tarm, analområde	1	1		
DB.01	Normalvariation. Magsäck, tarm, analområde				
DB.02	Symtom på sjukdom utan fastställd orsak. Magsäck, tarm, analområde	1	1		
DB.02.01	Vomitus	1	1		
DB.02.01.01	Blodig kräkning	1	1		1
DB.02.02	Diarré	1	1	1	
DB.02.02.01	Hemorragisk diarré, utan fastställd orsak	1	1	1	1
DB.02.03	Blod i avföringen	1	1		1
DB.02.03.01	Hematochezi	1	1		1
DB.02.03.02	Melena	1	1		1
DB.02.04	Dysfagi				
DB.02.05	Borborygmi				
DB.02.06	Flatulens				
DB.02.07	Abnormt luktande avföring				

DB.02.08	Tenesmus	1	1		
DB.02.09	Kolik	1	1		
DB.02.09.01	Gaskolik	1	1		
DB.02.09.02	Krampkolik	1	1		
DB.02.10	Koprostas				
DB.02.10.01	Magsäcksinpackning				
DB.02.10.01.01	Löpmagsinpackning				
DB.02.10.02	Koprostas. Ileum				
DB.02.10.03	Koprostas. Cekum				
DB.02.10.04	Koprostas. Colon				
DB.02.10.04.01	Koprostas. Distala colon				
DB.02.10.04.02	Koprostas. Rektum				
DB.02.10.04.03	Mekoniumförstoppning				
DB.03	Missbildningar, utvecklings-, tillväxtrubbningar. Magsäck, tarm, analområde				

DB.03.01	Missbildningar. Magsäck, tarm, analområde				
DB.03.01.01	Atresia ani/recti				
DB.03.01.02	Ileo-colon aganglionos				
DB.03.01.03	Meckels divertikulum				
DB.03.02	Utvecklings-, tillväxtrubbningar. Magsäck, tarm, analområde				
DB.03.02.01	Pylorusstenos				
DB.04	Metabola, nutritionella, degenerativa/dystrofiska förändringar. Magsäck, tarm, analområde				
DB.04.01	Metabola förändringar. Magsäck, tarm, analområde				
DB.04.01.01	Uremisk gastrit				
DB.04.01.02	Fölbrunstdiarré				
DB.04.01.03	Tympanism, sekundär kronisk				
DB.04.02	Nutritionella förändringar. Magsäck, tarm, analområde				

DB.04.02.01	Våmacidos				
DB.04.02.02	Våmalkalos				
DB.04.02.03	Trumsjuka				
DB.04.02.03.01	Tympanism, primär akut				
DB.04.02.03.02	Tympanism, primär kronisk				
DB.04.02.03.03	Tympanism, sekundär till obstruktion matstrupe				
DB.04.02.04	Nutritionellt orsakad diarré	1			
DB.04.03	Degenerativa/dystrofiska förändringar. Magsäck, tarm, analområde	1			
DB.05	Cirkulatoriska förändringar. Magsäck, tarm, analområde				
DB.05.01	Blödningar. Magsäck, tarm, analområde	1	1		1
DB.05.01.01	Blödning. Magsäck	1	1		1
DB.05.01.02	Blödning. Tarm	1	1		1

DB.05.01.02.01	Blödning. Tunntarm	1	1		1
DB.05.01.02.02	Blödning. Grovtarm	1	1		1
DB.05.02	Ödem. Magsäck, tarm, analområde				
DB.05.02.01	Ödem. Tarm				
DB.05.03	Infarkt. Magsäck, tarm, analområde				
DB.05.03.01	Ischemi. Tarm				
DB.05.04	Trombo/ emboli. Magsäck, tarm, analområde				
DB.05.04.01	Trombotisk-embolisk kolik				
DB.05.05	Arterio- och ateroskleros förändringar. Magsäck, tarm, analområde				
DB.06	Inflammatoriska, infektiösa förändringar. Magsäck, tarm, analområde	1	1		
DB.06.01	Inflammation. Magsäck, tarm, analområde	1	1		

DB.06.01.01	Inflammation, serös/serofibrinös. Magsäck, tarm, analområde	1	1		
DB.06.01.01.01	Inflammation, serös/serofibriös, akut. Magsäck, tarm, analområde	1	1		
DB.06.01.01.02	Inflammation, serös/serofibrinös, kronisk. Magsäck, tarm, analområde	1			
DB.06.01.02	Inflammation, purulent. Magsäck, tarm, analområde	1	1		
DB.06.01.02.01	Inflammation, purulent, akut. Magsäck, tarm, analområde	1	1		
DB.06.01.02.02	Inflammation, purulent, kronisk. Magsäck, tarm, analområde	1			
DB.06.01.03	Abscess/fistel. Magsäck, tarm, analområde				
DB.06.01.03.01	Tarmväggs- /krös abscess				

DB.06.01.03.02	Perirektal/perianal abscess				
DB.06.01.04	Gastrit	1	1		
DB.06.01.04.01	Gastrit, akut	1	1		
DB.06.01.04.01.01	Ulcerös gastrit, akut	1	1		
DB.06.01.04.01.02	Eosinofil gastrit, akut	1	1		
DB.06.01.04.01.03	Atrofisk gastrit, akut	1	1		
DB.06.01.04.01.04	Hypertrofisk gastrit, akut	1	1		
DB.06.01.04.01.05	Erosiv gastrit, akut	1	1		
DB.06.01.04.01.06	Follikulär gastrit, akut	1	1		
DB.06.01.04.02	Gastrit, kronisk	1			
DB.06.01.04.02.01	Ulcerös gastrit, kronisk	1			
DB.06.01.04.02.02	Eosinofil gastrit, kronisk	1			
DB.06.01.04.02.03	Atrofisk gastrit, kronisk	1			

DB.06.01.04.02.04	Hypertrofisk gastrit, kronisk	1			
DB.06.01.04.02.05	Erosiv gastrit, kronisk	1			
DB.06.01.04.02.06	Follikulär gastrit, kronisk	1			
DB.06.01.05	Rumenit				
DB.06.01.06	Gastroenterit	1	1	1	
DB.06.01.06.01	Gastroenterit, akut	1	1	1	
DB.06.01.06.01.01	Eosinofil gastroenterit, akut	1	1	1	
DB.06.01.06.02	Gastroenterit, kronisk	1			
DB.06.01.06.02.01	Eosinofil gastroenterit, kronisk	1			
DB.06.01.07	Gastroenterokolit	1	1	1	
DB.06.01.07.01	Eosinofil gastroenterokolit	1			
DB.06.01.08	Enterit	1	1	1	
DB.06.01.08.01	Enterit, akut	1	1	1	

DB.06.01.08.01.01	Lymfocytär enterit, akut	1	1	1	
DB.06.01.08.01.02	Lymfocytär-plasmacytär enterit, akut	1	1	1	
DB.06.01.08.01.03	Granulomatös enterit, akut	1	1	1	
DB.06.01.08.01.04	Eosinofil enterit, akut	1	1	1	
DB.06.01.08.01.05	Histiocytär enterit, akut	1	1	1	
DB.06.01.08.01.06	Ulcerös enterit, akut	1	1	1	
DB.06.01.08.01.07	Erosiv enterit, akut	1	1	1	
DB.06.01.08.01.08	Duodenit-proximal jejunit	1	1	1	
DB.06.01.08.02	Enterit, kronisk	1			
DB.06.01.08.02.01	Lymfocytär enterit, kronisk	1			
DB.06.01.08.02.02	Lymfocytär-plasmacytär enterit, kronisk	1			

DB.06.01.08.02.03	Granulomatös enterit, kronisk	1			
DB.06.01.08.02.04	Eosinofil enterit, kronisk	1			
DB.06.01.08.02.05	Histiocytär enterit, kronisk	1			
DB.06.01.08.02.06	Ulcerös enterit, kronisk	1			
DB.06.01.08.02.07	Erosiv enterit, kronisk	1			
DB.06.01.08.03	Kolit	1	1	1	
DB.06.01.08.03.01	Lymfocytär kolit	1			
DB.06.01.08.03.02	Lymfocytär-plasmacytär kolit	1			
DB.06.01.08.03.03	Eosinofil kolit	1			
DB.06.01.08.03.04	Histiocytär kolit	1			
DB.06.01.08.03.05	Histocytär ulcerös kolit	1			
DB.06.01.08.03.06	Ulcerös kolit	1			
DB.06.01.08.03.07	Erosiv kolit	1			

DB.06.01.09	Proktit	1	1		
DB.06.01.09.01	Proktit, akut	1	1		
DB.06.01.09.02	Proktit, kronisk	1			
DB.09	Traumatiska skador, främmande kroppar, lägesförändringar, termiska skador. Magsäck, tarm, analområde				
DB.09.01	Trauma. Magsäck, tarm, analområde				
DB.09.01.01	Ruptur. Magsäck				
DB.09.01.02	Vagusindigestion				
DB.09.01.02.01	Vasst				
DB.09.01.03	Kardiastenos				
DB.09.01.04	Krösruptur				
DB.09.01.05	Ruptur. Tarm				
DB.09.01.05.01	Tarmskada efter rektalisering				
DB.09.01.06	Trauma. Rektum				

DB.09.01.06.01	Rektovaginalfistel/ kloak				
DB.09.01.07	Tarmstriktur				
DB.09.01.07.01	Tunntarmsstriktur				
DB.09.01.07.02	Grovtarmsstriktur				
DB.09.01.07.03	Rektumstriktur				
DB.09.02	Främmande kroppar. Magsäck, tarm, analområde				
DB.09.02.01	Främmande kropp. Magsäck				
DB.09.02.02	Främmande kropp. Tunntarm				
DB.09.02.03	Främmande kropp. Colon/rektum				
DB.09.02.03.01	Sandkolik	1	1		
DB.09.03	Lägesförändringar. Magsäck, tarm, analområde				
DB.09.03.01	Adherenser mellan tarm/magsäck/övriga dig.organ				

DB.09.03.02	Lägeförändring. Magsäck				
DB.09.03.02.01	Magsäcksdilatation				
DB.09.03.02.02	Magomvridning				
DB.09.03.02.03	Magsäcksdilatation och magomvridning				
DB.09.03.02.04	Löpmagsdislokation/dilatation				
DB.09.03.02.04.01	Vänstersidig löpmagsdislokation/dilatation				
DB.09.03.02.04.02	Vänstersidig löpmagsdislokation med torsion				
DB.09.03.02.04.03	Högersidig löpmagsdislokation/dilatation				
DB.09.03.02.04.04	Högersidig löpmagsdislokation med torsion				
DB.09.03.02.05	Framfall av magsäck i esofagus				

DB.09.03.03	Lägesförändring. Tarm				
DB.09.03.03.01	Tarmomvridning				
DB.09.03.03.01.01	Tunntarmsvolvolus				
DB.09.03.03.01.02	Omvridning av grovtarm				
DB.09.03.03.01.03	Flexion av grovtarm				
DB.09.03.04	Invagination				
DB.09.03.04.01	Invagination av tunntarm				
DB.09.03.04.01.01	Invagination av ileum i colon				
DB.09.03.04.02	Invagination av cekum				
DB.09.03.04.03	Invagination av colon				
DB.09.03.05	Inkarceration av tarm				
DB.09.03.05.01	Inkarceration i foramen epiploicum				

DB.09.03.05.02	Inkarceration av tarm i pungbråck				
DB.09.03.05.03	Inkarceration av tarm i navelbråck				
DB.09.03.05.04	Upphängning av grovtarm på mjält-njurbandet				
DB.09.03.06	Tarmframfall				
DB.09.03.06.01	Rektumprolaps				
DB.09.03.07	Pendulerende kröslipom med sekundär avsnörning av tarm				
DB.09.04	Termiska skador. Magsäck, tarm				
DB.10	Toxiska förändringar. Magsäck, tarm, analområde	1	1		
DB.11	Idiopatiska, ospecifika, multifaktoriella tillstånd. Magsäck, tarm, analområde	1	1		
DB.11.01	Refluxgastrit	1	1		
DB.11.02	Magsår/löpmagssår	1	1		
DB.11.02.01	Blödande magsår	1	1		

DB.11.03	Ulcus i tunntarm	1	1		
DB.11.04	Malabsorption				
DB.11.05	Protein-loosing enteropati	1			
DB.11.05.01	Intestinal lymfangiektasi	1			
DB.11.06	Tarmflorerubbning	1	1	1	
DB.11.07	Krävdilatation				
DB.11.08	Funktionell ileus				
DB.11.09	Indigestion				
DE	Exokrin pankreas				
DE.01	Normalvariation. Exokrina pankreas				
DE.02	Symtom på sjukdom utan fastställd orsak. Exokrin pankreas				
DE.02.01	Exokrin pankreasinsufficiens				
DE.03	Missbildningar, utvecklings-, tillväxtrubbningar. Exokrin pankreas				
DE.03.01	Missbildningar. Exokrina pankreas				
DE.03.02	Utvecklings-, tillväxtrubbningar. Exokrina pankreas				

DE.04	Metabola, nutritionella, degenerativa/dystrofiska förändingar. Exokrina pankreas				
DE.04.01	Metabola förändringar. Exokrina pankreas				
DE.04.02	Nutritionella förändringar. Exokrina pankreas				
DE.04.03	Degenerativa/dystrofiska förändringar. Exokrina pankreas				
DE.05	Cirkulatoriska förändringar. Exokrina pankreas				
DE.05.01	Blödningar. Exokrina pankreas				
DE.05.02	Ödem. Exokrina pankreas				
DE.05.03	Infarkter. Exokrina pankreas				
DE.05.04	Trombos/emboli. Exokrina pankreas				
DE.05.05	Arterio- och aterosklerosförändringar. Exokrina pankreas				
DE.06	Inflammatoriska, infektiösa förändringar. Exokrina pankreas				
DE.06.01	Pankreatit				

DE.06.01.01	Pankreatit, serös/serofibrinös				
DE.06.01.01.01	Pankreatit, serös/serofibrinös, akut				
DE.06.01.01.02	Pankreatit, serös/serofibrinös, kronisk				
DE.06.01.02	Pankreatit, purulent				
DE.06.01.02.01	Pankreatit, purulent, akut				
DE.06.01.02.02	Pankreatit, purulent, kronisk				
DE.06.01.03	Abscess/fistel. Exokrina pankreas				
DE.06.02	Parasitära infektioner. Exokrina pankreas				
DE.07	Immunmedierade tillstånd. Exokrina pankreas				
DE.09	Traumatiska skador, främmande kroppar, lägesförändringar, termiska skador. Exokrina pankreas				
DE.09.01	Traumatiska skador. Exokrina pankreas				

DE.09.02	Främmande kroppar. Exokrina pankreas				
DE.09.03	Lägesförändringar. Exokrina pankreas				
DE.10	Toxiska förändringar. Exokrina pankreas				
DE.11	Idiopatiska, ospecifika, multifaktoriella tillstånd. Exokrina pankreas				
DB	Magsäck, tarm, analområde	1	1		
DB0	Normalvar, symtom u fastställd orsak, magsäck tarm	1	1		
DB00	Normalvariation, magsäck tarm				
DB01	Symtom på sjukdom u fastställd orsak, magsäck tarm	1	1		
DB011	Kräkning	1	1		
DB0111	Blodig kräkning	1	1		1
DB012	Symtom på magsmärtor				
DB013	Diarré	1	1	1	
DB0131	Blodig diarré	1	1	1	1
DB014	Kräkning och diarré	1	1	1	
DB0141	Blodig kräkning och diarré	1	1	1	1

DB015	Abnormt luktande avföring				
DB016	Tenesmus	1	1		
DB017	Symtom på förstoppning				
DB018	Kolik	1	1		
DB0181	Kolik u fastställd orsak	1	1		
DB0182	Gaskolik	1	1		
DB0183	Krampkolik	1	1		
DB1	Missbildn, utveckl. rubbn, tillväxtrubbn, magsäck tarm				
DB10	Missbildning, magsäck tarm				
DB101	Atresia ani/recti				
DB102	Ileo-colon agangnlionos (letalt vitt föl)				
DB11	Utvecklingsrubbn, tillväxtrubbn, magsäck tarm				
DB111	Pylorusstenos				
DB2	Metabol, nutrit, degen/dystrof förändr, magsäck tarm				
DB20	Metaboliska förändringar, magsäck tarm				

DB201	Uremisk gastrit				
DB202	Fölbrunstdiarré / nio dagars diarré				
DB203	Utfodringsbetingad diarré				
DB204	Kronisk sekundär tympanism (trumsjuka pga ruminit mellankalv				
DB21	Nutritionella förändringar, magsäck tarm				
DB211	Våmacidos				
DB212	Akut primär tympanism (trumsjuka, bete)				
DB213	Kronisk primär typmanism (trumsjuka, stall)				
DB22	Degenerativa/dystrofiska förändringar, magsäck tarm	1			
DB23	Hormonella förändringar, magsäck tarm				
DB3	Cirkulatoriska förändringar, magsäck tarm				
DB30	Blödningar, magsäck tarm	1	1		1
DB301	Blödning från magsäck	1	1		1

DB302	Blödning till tarm	1	1		1
DB31	Ödem, magsäck tarm	1	1		
DB311	Ödem i tarmvägg	1	1		
DB32	Infarkter, magsäck tarm				
DB321	Ischemi i tarmavsnitt				
DB34	Trombos / emboli, magsäck tarm				
DB341	Trombotisk-embolisk kolik				
DB35	Arterio-atero-skleros förändringar, magsäck tarm				
DB4	Infekt, infl förändringar, magsäck tarm	1	1		
DB40	Specifika infektionssjukdomar, magsäck tarm	1	1		
DB401	Spädgrisdiarré				
DB41	Akuta inflammationstillstånd, magsäck tarm	1	1		
DB411	Akut serös / serofibrinös inflammation, magsäck tarm	1	1		
DB412	Akut purulent inflammation, magsäck tarm	1	1		

DB413	Specifika akuta infl tillstånd, magsäck tarm	1	1		
DB419	Övriga akuta infl tillstånd, magsäck tarm	1	1		
DB4191	Akut gastrit	1	1		
DB4192	Akut enterit	1	1	1	
DB41921	Akut främre enterit	1	1	1	
DB4193	Akut gastroenterit	1	1	1	
DB4194	Akut kolit / tyflit	1	1	1	
DB4195	Akut proktit	1	1		
DB42	Kroniska inflammationstillstånd, magsäck tarm	1			
DB421	Kronisk serös inflammation, magsäck tarm	1			
DB422	Purulent inflammation, magsäck tarm	1			
DB423	Abscess/fistel, magsäck tarm				
DB4231	Tarmväggs- / krös abscess				
DB4232	Perirektal / perianal abscess				
DB424	Specifika kroniska infl tillstånd, magsäck tarm	1			

DB4241	Kronisk eosinofil gastrit /enterit /gastroenterit	1			
DB4242	Kronisk lymfocytär enterit	1			
DB4243	Kronisk lymfocytär-plasmacytär enterit	1			
DB4244	Kronisk granulomatös enterit	1			
DB4245	Kronisk eosinofil kolit	1			
DB4247	Kronisk granulomatös kolit	1			
DB429	Övriga kroniska infl tillstånd, magsäck tarm	1			
DB4290	Ruminit				
DB4291	Kronisk gastrit	1			
DB42911	Kronisk atrofisk gastrit	1			
DB42912	Kronisk hypertrofisk gastrit	1			
DB42913	Kronisk erosiv gastrit (inklusive ulkus)	1			
DB42914	Kronisk follikulär gastrit	1			
DB4292	Kronisk enterit	1			
DB4293	Kronisk kolit /tyflit	1			
DB42931	Kronisk ulcerös / erosiv kolit	1			

DB42932	Colon irritabele	1			
DB4294	Kronisk proktit	1			
DB4295	Kronisk analsäcksinflammation				
DB43	Parasitära sjukdomar, magsäck tarm	1	1		
DB5	Immunmedierade tillstånd, magsäck tarm	1			
DB6	Neoplastiska förändringar, magsäck tarm				
DB7	Trauma, fr kr, lägeförändr, term skad, magsäck tarm				
DB71	Traumatiska / mekaniska skador, magsäck tarm				
DB711	Magsäcksdilatation efter foderförätning				
DB712	Förstoppning				
DB7121	Förstoppning i tunntarm				
DB7122	Förstoppning i cekum (blindtarm)				
DB7123	Förstoppning i kolon (grovtarm)				
DB7124	Förstoppning i lilla kolon				

DB7125	Förstoppning i rektum (ändtarm)				
DB7126	Mekoniumförstoppning				
DB713	Kardiastenos				
DB714	Ruptur av magsäck				
DB715	Ruptur av tarm				
DB716	Tarmstrikturer				
DB7161	Rektumstriktur				
DB717	Krösruptur				
DB718	Rektumskador				
DB7181	Perforation efter rektalisering				
DB719	Rektovaginalfistel / kloak				
DB72	Främmande kroppar, magsäck tarm				
DB721	Främmande kropp i magsäck				
DB722	Främmande kropp i tunntarm				
DB723	Främmande kropp i kolon / rektum				
DB724	Sandinpackning				
DB725	Främmande kropp i analområdet				

DB726	Traumatisk retikuloperitonit (vasst)				
DB728	Löpmagsinpackning				
DB729	Vagusindigestion (vagusskada vid vasst")"				
DB73	Lägesförändringar, magsäck tarm				
DB731	Löpmagsomvridning UNS (löpmagsdislokation UNS)				
DB7311	Magsäcksdilatation				
DB7312	Magsäcksomvridning				
DB7313	Framfall av magsäck i esofagus				
DB7314	Vänstersidig löpmagsdislokation				
DB7315	Högersidig löpmagsdislokation				
DB732	Tarmlägesförändring				
DB7321	Omvridning av tunntarm				
DB7322	Omvridning av grovtarm				
DB733	Invagination				
DB7331	Invagination av tunntarm				

DB7332	Invagination av cekum				
DB7333	Invagination av kolon				
DB734	Inkarceration av tarm				
DB7341	Inkarceration i foramen epiploicum				
DB7342	Inkarceration av tarm i pungbråck				
DB735	Tarmframfall				
DB7351	Tarmframfall efter kastration				
DB7352	Ändtarmsframfall				
DB736	Striktur av tarm pga stjälkat lipom				
DB74	Termiska skador, magsäck tarm				
DB8	Toxiska förändringar, magsäck tarm	1	1		
DB9	Idiopatiska, ospecif, multifakt, magsäck tarm	1	1		
DB91	Refluxgastrit	1	1		
DB92	Ulcus ventriculi, magsår / löpmagssår	1	1		
DB93	Malabsorption				
DB931	Neonatal steatorré (Rotav./Coccidier/stress sk.3-v diarré)				

DB932	Avvänjningsdiarré svin				
DB94	Protein-loosing enteropati	1			
DB941	Lymfangiektasi	1			
DB95	Tarmflorerubbning	1			
DB96	Krävdilatation (hängkräva)				
DB97	Ileus				
DE	Pankreas				
DE0	Normalvar, symtom u fastställd orsak, pankreas				
DE00	Normalvariation, pankreas				
DE01	Symtom på sjukdom u fastställd orsak, pankreas				
DE1	Missbildn, utveckl. rubbn, tillväxtrubbn, pankreas				
DE10	Missbildning, pankreas				
DE11	Utvecklingsrubbn, tillväxtrubbn, pankreas				
DE2	Metabol, nutrit, degen/dystrof förändr, pankreas				

DE20	Metaboliska förändringar, pankreas				
DE21	Nutritionella förändringar, pankreas				
DE22	Degenerativa/dystrofiska förändringar, pankreas				
DE23	Hormonella förändringar, pankreas				
DE3	Cirkulatoriska förändringar, pankreas				
DE30	Blödningar, pankreas				
DE31	Ödem, pankreas				
DE32	Infarkter, pankreas				
DE34	Trombos / emboli, pankreas				
DE35	Arterio-atero-skleros förändringar, pankreas				
DE4	Infekt, infl förändringar, pankreas				
DE40	Specifika infektionssjukdomar, pankreas				
DE41	Akuta inflammationstillstånd, pankreas				

DE411	Akut serös / serofibrinös inflammation, pankreas				
DE412	Akut purulent inflammation, pankreas				
DE413	Specifika akuta infl tillstånd, pankreas				
DE419	Övriga akuta infl tillstånd, pankreas				
DE4191	Akut pankreatit				
DE42	Kroniska inflammationstillstånd, pankreas				
DE421	Kronisk serös inflammation, pankreas				
DE422	Purulent inflammation, pankreas				
DE423	Abscess/fistel, pankreas				
DE424	Specifika kroniska infl tillstånd, pankreas				
DE429	Övriga kroniska infl tillstånd, pankreas				
DE4291	Kronisk intermittent pankreatit				
DE43	Parasitära sjukdomar, pankreas				

DE5	Immunmedierade tillstånd, pankreas				
DE613	Zollinger-Ellison syndromet				
DE7	Trauma, fr kr, lägeförändr, term skad, pankreas				
DE71	Traumatiska / mekaniska skador, pankreas				
DE72	Främmande kroppar, pankreas				
DE73	Lägesförändringar, pankreas				
DE8	Toxiska förändringar, pankreas				
DE9	Idiopatiska, ospecif, multifakt, pankreas				
DE91	Exokrin pankreasinsufficiens				
