# Supplementary material for: How low can you go? Antibiotic use in Swedish dogs with gastroenteritis
Source: Front Vet Sci. 2024 Dec 18;11:1506106. doi: 10.3389/fvets.2024.1506106 (PMC11688813; doi:10.3389/fvets.2024.1506106)
Supplement: SUPPLEMENTARY DATA SHEET 2 — Number of CGE consultations (including subcategories) and level of antibiotic use per year 2020–2023. [file Data_Sheet_2.PDF]

	**All CGE**	**ACGE**	**NDACGE**	**DACGE**	**Non-hemorrhagic DACGE**	**Hemorrhagic DACGE**	**CCGE**
**Number of consultations**	21799	19635	7126	12509	10026	2483	2164
Non-hospitalized	17562	15474	6017	9457	7972	1485	2088
Hospitalized	4237	4161	1109	3052	2054	998	76
**Antibiotic use, all dogs *n* (%)**	*1775 (8.14%)*	*1724 (8.78%)*	*318 (4.46%)*	*1406 (11.24%)*	*750 (7.48%)*	*656 (26.42%)*	*51 (2.36%)*
Non-hospitalized	*602 (3.43%)*	*571 (3.69%)*	*118 (1.96%)*	*453 (4.79%)*	*267 (3.35%)*	*186 (12.53%)*	*31 (1.48%)*
Hospitalized	*1173 (27.68%)*	*1153 (27.71%)*	*200 (18.03%)*	*953 (31.23%)*	*483 (23.52%)*	*470 (47.09%)*	*20 (26.32%)*

Number of CGE consultations with subcategories and antibiotic use during year 2020.

	**All CGE**	**ACGE**	**NDACGE**	**DACGE**	**Non-hemorrhagic DACGE**	**Hemorrhagic DACGE**	**CCGE**
**Number of consultations**	23423	21656	8689	12967	11642	1325	1767
Non-hospitalized	18753	17070	7379	9691	8927	764	1683
Hospitalized	4670	4586	1310	3276	2715	561	84
**Antibiotic use, all dogs *n (%)***	*1426 (6.09%)*	*1393 (6.43%)*	*338 (3.89%)*	*1055 (8.14%)*	*768 (6.60%)*	*287 (21.66%)*	*33 (1.87%)*
Non-hospitalized	*456 (2.43%)*	*443 (2.6%)*	*133 (1.80%)*	*310 (3.2%)*	*234 (2.62%)*	*76 (9.95%)*	*133 (1.80%)*
Hospitalized	*970 (20.77%)*	*950 (20.72%)*	*205 (15.65%)*	*745 (22.74%)*	*534 (19.67%)*	*211 (37.61%)*	*20 (23.81%)*

Number of CGE consultations with subcategories and antibiotic use during year 2021.

	All CGE	ACGE	NDACGE	DACGE	Non-hemorrhagic DACGE	Hemorrhagic DACGE	CCGE
**Number of consultations**	23381	21599	8766	12833	11265	1568	1782
Non-hospitalized	19097	17381	7495	9886	8976	910	1716
Hospitalized	4284	4218	1271	2947	2289	658	66
**Antibiotic use, all dogs *n* (%)**	*1160 (4.96%)*	*1124 (5,2%)*	*270 (3.08%)*	*854 (6.65%)*	*543 (4.82%)*	*311 (19.83%)*	*36 (2.02%)*
Non-hospitalized	*278 (1.46%)*	*262 (1.51%)*	*73 (0.97%)*	*189 (1.91%)*	*133 (1.48%)*	*56 (6.15%)*	*16 (0.93%)*
Hospitalized	*882 (20.59%)*	*862 (20.44%)*	*197 (15.50%)*	*665 (22.57%)*	*410 (17.91%)*	*225 (38.75%)*	*20 (30.30%)*

Number of CGE consultations with subcategories and antibiotic use during year 2022.

	All CGE	ACGE	NDACGE	DACGE	Non-hemorrhagic DACGE	Hemorrhagic DACGE	CCGE
**Number of consultations**	25038	23153	9038	14115	12301	1814	1885
Non-hospitalized	21024	19213	7950	11263	10135	1128	1811
Hospitalized	4014	3940	1088	2852	2166	686	74
**Antibiotic use, all dogs *n* (%)**	*970 (3.87%)*	*949 (4.1%)*	*262 (2.90%)*	*687 (4.87%)*	*431 (3.5%)*	*256 (14.11%)*	*21 (1.11%)*
Non-hospitalized	*250 (1.19%)*	*246 (1.28%)*	*82 (1.03%)*	*164 (1.46%)*	*118 (1.16%)*	*46 (4.08%)*	*4 (0.22%)*
Hospitalized	*720 (17.94%)*	*703 (17.84%)*	*180 (16.54%)*	*523 (18.34%)*	*313 (14.45%)*	*210 (30.61%)*	*17 (22.97%)*

Number of CGE consultations with subcategories and antibiotic use during year 2023.

CGE: canine gastroenteritis

ACGE: acute canine gastroenteritis

NDACGE: Non-diarrhetic acute canine gastroenteritis

DACGE: Diarrhetic acute canine gastroenteritis

CCGE: Chronic canine gastroenteritis
