# Supplementary material for: How low can you go? Antibiotic use in Swedish dogs with gastroenteritis
Source: Front Vet Sci. 2024 Dec 18;11:1506106. doi: 10.3389/fvets.2024.1506106 (PMC11688813; doi:10.3389/fvets.2024.1506106)
Supplement: SUPPLEMENTARY DATA SHEET 3 — Antibiotic substances used in CGE consultations (including subcategories) year 2020–2023. [file Data_Sheet_3.PDF]

Year	Aminopenicillins (%)	Metronidazole (%)	Quinolones (%)	Potentiated sulphonamides (%)	Tetracyclines (%)	Lincosamides (%)
2020	54.6	51.4	3.3	2.1	1.3	0.1
2021	60.5	43.1	2.4	2.3	2	0.1
2022	62.2	39.5	3.8	3.9	1.9	1
2023	67.5	33.3	5.1	3.9	2.2	0.1
2020-2023	60.6	43.3	3.5	2.9	1.8	0.9

Antibiotic substances used in all CGE consultations 2020-2023 (%).

Year	Aminopenicillins (%)	Metronidazole (%)	Quinolones (%)	Potentiated sulphonamides (%)	Tetracyclines (%)	Lincosamides (%)
2020	55.2	51.5	3.3	2.1	1.3	0.1
2021	60.9	43.1	2.3	2.4	1.9	0.9
2022	62.5	39.7	3.4	3.9	1.8	0.1
2023	67.8	33.4	5.2	3.9	2.1	0.6
2020-2023	50.6	43.4	3.4	2.9	1.7	0.8

Antibiotic substances used in ACGE 2020-2023 (%).

Year	Aminopenicillins (%)	Metronidazole (%)	Quinolones (%)	Potentiated sulphonamides (%)	Tetracyclines (%)	Lincosamides (%)
2020	80.5	19.18	5.66	3.46	2.52	0.94
2021	81.65	12.13	2.66	3.55	4.73	0.89
2022	82.59	8.15	2.59	10.37	14.81	1.11
2023	80.92	8.4	3.05	5.25	4.2	1.15
2020-2023	81.28	12.39	3.54	5.9	3.29	1.01

Antibiotic substances used in NDACGE 2020-2023 (%).

Year	Aminopenicillins (%)	Metronidazole (%)	Quinolones (%)	Potentiated sulphonamides (%)	Tetracyclines (%)	Lincosamides (%)
2020	67.71	58.75	2.77	1.78	1	0.5
2021	54.22	52.99	2.18	1.99	0.95	0.95
2022	56.09	49.65	3.63	1.87	1.87	0.94
2023	62.74	29.89	5.97	2.62	1.31	0.44
2020-2023	54.44	52.54	3.33	2	1.23	0.7

Antibiotic substances used in DACGE 2020-2023 (%).

Year	Aminopenicillins (%)	Metronidazole (%)	Quinolones (%)	Potentiated sulphonamides (%)	Tetracyclines (%)	Lincosamides (%)
2020	54.27	50	2.53	2.13	1.87	0.93
2021	55.73	48.7	1.95	2.34	1.04	1.17
2022	57.83	42.17	2.76	2.39	2.95	1.29
2023	67.29	35.5	4.64	3.94	1.86	0.7
2020-2023	57.79	45.34	2.77	2.57	1.85	1.04

Antibiotic substances used in non-haemorrhagic DACGE 2020-2023 (%).

Year	Aminopenicillins (%)	Metronidazole (%)	Quinolones (%)	Potentiated sulphonamides (%)	Tetracyclines (%)	Lincosamides (%)
2020	44.05	68.75	3.05	1.37	0	0
2021	50.17	64.46	2.79	1.05	0.7	0.35
2022	53.05	62.7	5.14	0.96	0	0.32
2023	55.08	55.45	8.2	0.39	0.39	0
2020-2023	48.91	64.43	4.25	1.06	0.2	0.13

Antibiotic substances used in haemorrhagic DACGE 2020-2023 (%).

Year	Aminopenicillins (%)	Metronidazole (%)	Quinolones (%)	Potentiated sulphonamides (%)	Tetracyclines (%)	Lincosamides (%)
2020	33.33	50.98	3.92	3.92	1.96	7.84
2021	45.45	45.45	6.06	0	3.03	3.03
2022	55.55	33.33	16.67	2.78	5.55	0
2023	57.14	28.57	0	4.76	4.76	9.52
2020-2023	45.71	41.43	7.14	2.86	3.57	5

Antibiotic substances used in CCGE 2020-2023 (%).

CGE: canine gastroenteritis
ACGE: acute canine gastroenteritis
NDACGE: Non-diarrhoetic acute canine gastroenteritis
DACGE: Diarrhoetic acute canine gastroenteritis
CCGE: Chronic canine gastroenteritis
